# Dr. Lucien La Bland Brainard

**Published:** 1914-01

**Authors:** 


### OBITUARIES.

*Readers are requested to report promptly the death of all physicians in Western New York, or former residents of this region, or graduates of any medical school in Western New York and to notify the families of the deceased of our desire to publish adequate obituary notices.*

Dr. Lucien La Bland Brainard, N. Y. Homeopathic Medical College, 1874, died suddenly, Nov. 18, of heart failure at his home in Little Falls, aged 60. He was Vice President of the local National Bank and was formerly on the consulting staff of the Faxton Hospital of Utica.

